# The blood–brain barrier in systemic infection and inflammation

**DOI:** 10.1038/s41423-021-00757-x

**Published:** 2021-09-30

**Authors:** Ian Galea

**Affiliations:** Clinical Neurosciences, Clinical & Experimental Sciences, Faculty of Medicine, University of Southampton, Southampton, SO16 6YD UK

**Keywords:** blood–brain barrier, inflammation, infection, signaling, moderation, Sepsis, Neuroimmunology

## Abstract

The vascular blood–brain barrier is a highly regulated interface between the blood and brain. Its primary function is to protect central neurons while signaling the presence of systemic inflammation and infection to the brain to enable a protective sickness behavior response. With increasing degrees and duration of systemic inflammation, the vascular blood–brain barrier becomes more permeable to solutes, undergoes an increase in lymphocyte trafficking, and is infiltrated by innate immune cells; endothelial cell damage may occasionally occur. Perturbation of neuronal function results in the clinical features of encephalopathy. Here, the molecular and cellular anatomy of the vascular blood–brain barrier is reviewed, first in a healthy context and second in a systemic inflammatory context. Distinct from the molecular and cellular mediators of the blood–brain barrier’s response to inflammation, several moderators influence the direction and magnitude at genetic, system, cellular and molecular levels. These include sex, genetic background, age, pre-existing brain pathology, systemic comorbidity, and gut dysbiosis. Further progress is required to define and measure mediators and moderators of the blood–brain barrier’s response to systemic inflammation in order to explain the heterogeneity observed in animal and human studies.

## Introduction

The brain is a delicate organ because neurons require a highly specialized environment to function properly. Ionic homeostasis of brain interstitial fluid [1] is essential to enable the maintenance and controlled modulation of transmembrane gradients and movements; these are necessary to generate accurate changes in electrical potential locally in synapses, along axons, or in distributed networks. The interstitial protein concentration is kept low [1] to minimize cellular proliferation and protein binding of charged ions and neurotransmitters, and to optimize the rheology of extracellular fluids at low pressures in the intracranial compartment. Leukocyte entry is highly regulated [2], with complete suppression of innate immune cell infiltration to avoid acute inflammatory responses in situ, since if neuronal damage occurs, regeneration is slow and limited. On the other hand, a low level of T-cell immunosurveillance is allowed to keep latent viruses under check with minimal inflammatory consequences.

The vascular blood–brain barrier (BBB) represents the brain’s main interface with its external environment, at which these processes are mostly controlled [2, 3]. Although referred to as a “barrier”, this is a misnomer since cells and substances can be exchanged bidirectionally [4, 5]. There are two main reasons why the barrier cannot be absolute. First, due to its high level of specialization, the brain depends on the rest of the body for the supply of nutrients and clearance of toxic byproducts of metabolism. For instance, the brain’s capacity for gluconeogenesis is limited [6], and glucose supply is heavily reliant on its transport across the vascular BBB via the insulin-independent glucose transporter GLUT-1 [7]. Second, during systemic inflammation, a number of brain responses occur that have survival value, collectively referred to as sickness behavior [8]. This is a set of coordinated physiological and behavioral changes orchestrated by the brain that protects the individual from predators while they are ill (such as via lethargy), enables them to fight the infection (such as via fever and anorexia), and protects the species as a whole (such as via anhedonia and social withdrawal). This permissive quality of the vascular BBB may sometimes be taken too far during sustained or overwhelming systemic inflammation or in cases where there is already BBB damage due to neurological disease [9].

**Fig. 1:**
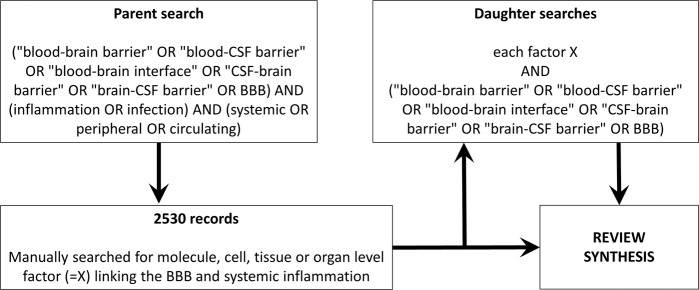
Review methodology. A stepwise semi-automated approach was taken: an automated parent search was followed by a manual step, which then led to multiple daughters automated searches. The searches were last updated on July 18, 2021

This review focuses on current knowledge regarding the relationship between systemic inflammation and the vascular BBB, with three aims: (1) to describe the molecular and cellular components of the BBB that may respond to inflammation, (2) to review how the BBB responds to systemic inflammation, and (3) to define those factors that are known to moderate this relationship. The literature search strategy employed is illustrated in Fig. 1. In summary, a multistep approach was used. If the parent search revealed a molecule-, cell-, tissue-, or organ-level factor linking the BBB and systemic inflammation, the factor was searched separately. Due to the vast number of publications in this area, it was impossible to acknowledge all publications in this review. Lack of mention is not a judgment of study quality, since examples are used to illustrate specific key concepts.

Although this review discusses in detail the vascular BBB during systemic inflammation, it is important to place the vascular BBB in the context of other interfaces of the brain with its external environment [5]. These other interfaces arise since the central nervous system is highly compartmentalized. Their anatomical locations are listed in Table 1. While each of these other interfaces is likely to be important during systemic inflammation, they are less well studied than the vascular BBB in this setting, and their precise contributions remain to be determined. The vascular BBB has the largest surface area among the various interfaces, corresponding to a total surface area of 15–25 m^2^  [10], and will be hereafter referred to simply as the BBB.

**Table 1:** The blood–brain barrier in context

Interface	Anatomical locations
Blood–brain barrier [4]	Brain capillary walls
Blood–cerebrospinal fluid (CSF) interface [163]	Choroid plexus, arachnoid granulations, and leptomeningeal blood vessel walls
Circumventricular organ–brain interface [164]	Tanycytes at the junctions between circumventricular organs and the rest of the brain parenchyma
Systemic lymph–brain interface [165, 166]	Peri-arterial mural drainage pathway, cranial and spinal nerves, cribriform plate, and meninges
Viscera–brain interface [167]	Neural pathways such as the vagus nerve linking the central nervous system with the viscera

Interfaces of the brain with its external environment and their neuroanatomical locations

## BBB molecular and cellular anatomy

**Fig. 2:**
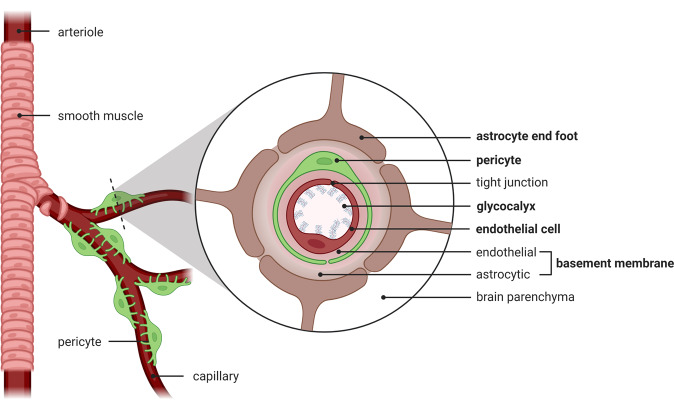
Vascular blood–brain barrier. Alternating layers of molecular (glycocalyx, basement membrane) and cellular (endothelial cells, pericytes, astrocytes) components form the blood–brain barrier. The five components are in bold typeface. The basement membrane is a single structure with a two-layer composition, since it is secreted by endothelial cells and astrocytes on either side

The BBB is composed of several components: the glycocalyx, endothelial cells, basement membrane containing pericytes, and astrocytic end-feet (Fig. 2). Some components are entirely molecular (the glycocalyx and basement membrane), while others are cellular (endothelial cells, pericytes, and astrocytes). This section will lay out how the alternating molecular and cellular layers exert control over solute and cellular transport across the BBB in different ways in the healthy state and during systemic inflammation.

### Glycocalyx

**Fig. 3:**
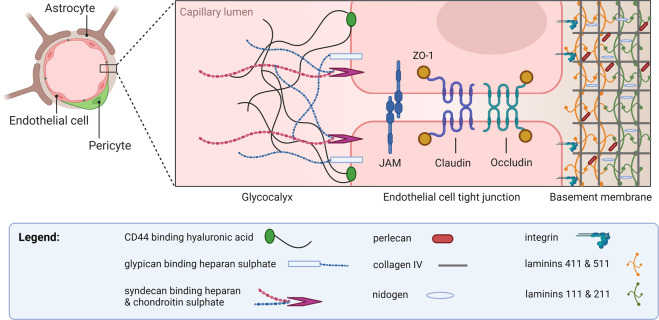
Molecular components of the BBB. The glycocalyx coats the luminal surface of cerebral endothelial cells, protruding into the lumen in a frond-like manner. The basement membrane is laid down by endothelial cells and astrocytes. While the two types of the extracellular matrix are biochemically distinct, they are indistinguishable and fused into one entity during health, only to be separated when perivascular spaces develop as a result of the accumulation of cells or fluid. JAM: junctional adhesion molecule

The glycocalyx, which is secreted by endothelial cells and coats the luminal surface of blood vessels, consists of a closely knitted network of glycosaminoglycans (heparan sulfate, chondroitin sulfate, keratan sulfate, and hyaluronic acid) (Fig. 3) [11, 12]. It is tethered to endothelial cells via membrane-associated CD44 and the proteoglycans syndecan and glypican (Fig. 3) [11, 12]. It has the appearance of a nonuniform thick bed of seaweed on the seafloor [11]. Orosomucoid secreted by endothelial cells [13] imparts a negative charge to the glycocalyx, which has been demonstrated to inhibit BBB permeability to negatively charged plasma proteins [14]. However, its main barrier function appears to be that of a molecular sieve, with a measurable 50% drop in the diffusion of molecular tracers of size 40 kD or above across its thickness [15]. The glycocalyx in the brain presents a more formidable barrier than the glycocalyx elsewhere in the body. One study showed 40% capillary luminal surface coverage in the brain, compared to 15% in the heart and 4% in the lungs; the thickness was also greatest in the brain (301 nm versus 136 nm in the heart, and 65 nm in the lungs) [16]. In addition, the glycocalyx in cerebral capillaries appears to be less permeable to 70-kD dextran [17], in contrast to the systemic glycocalyx [18].

Systemic inflammation in vivo leads to cerebral endothelial glycocalyx damage with reductions in thickness and coverage, as shown by several studies after intravenous [17] or intraperitoneal [16] lipopolysaccharide (LPS) challenge in mice. LPS is a proinflammatory component of bacterial cell walls used to model systemic inflammation in laboratory settings. Exposure to LPS, the cytokine tumor necrosis factor-α, or the procoagulant enzyme thrombin alone led to significant reductions in glycocalyx thickness in a human pulmonary microvascular endothelial cell line in vitro; [19] thus, several circulating molecules during systemic infection or inflammation can result in glycocalyx shedding. Additionally, enzymes from the family of matrix metalloproteinases (MMPs) secreted by rolling leukocytes or endothelial cells [20] may degrade glycocalyx components such as syndecan, as shown in human and mouse cell lines [21] and rat mesenteric vessels [22]. The glycocalyx also presents some obstruction to the interaction between leukocytes and the endothelial cell surface, which is needed for rolling and adhesion. This physical distancing is not sufficiently mitigated by the combined length of leukocyte pseudopod projections, the leukocyte selectin or integrin and their respective endothelial ligands (P-selectin glycoprotein 1 and intercellular adhesion molecules). Hence, glycocalyx degradation enhances leukocyte immobilization, as shown in mouse cremaster venules [23], which should facilitate entry into the tissue.

### Endothelial cells

Microcerebrovascular endothelial cells are specialized compared to their counterparts outside the brain [24]. They lack fenestrae, have tight junctions, exhibit low rates of macropinocytosis and caveolar transcytosis [25], express low levels of leukocyte adhesion molecules [9], and possess a plethora of substrate-specific transport systems that control the influx of polar nutrients into the brain (such as solute carrier, or SLC, family transporters) [26] and efflux of unwanted substances into the blood (such as ATP-binding cassette, or ABC, family transporters, e.g., P-glycoprotein) [26]. Several saturable transport systems have been predicted based on kinetic modeling of blood-to-brain transfer, yet their molecular identities remain unknown; an important example is the insulin transporter [27]. Tight junctions are composed of complexes of three types of molecules: occludins, claudins, and junctional adhesion molecules (Fig. 3). These molecular complexes form a continuous band around the capillary, and this circumferential molecular assembly constitutes a formidable barrier to small molecules diffusing down concentration gradients across the paracellular route. The size selectivity of tight junctions has been beautifully illustrated in claudin-5 knockout mice, which, compared to wild-type mice, showed increased permeability to 562 daltons but not 1.9-kD tracers [28]. Tight junctions are attached to the endothelial cytoskeleton by zonula occludens proteins; they are not static structures but rather respond to the physiological state of the endothelial cell and its environment, including systemic inflammation, as discussed further below. Compared to endothelial cells elsewhere, microcerebrovascular endothelial cells express lower levels of molecules involved in the transmigration of leukocytes into the brain; this includes E- and P-selectins, which mediate rolling, and the integrin ligands ICAM-1, PECAM-1, and VCAM-1, which mediate firm adhesion [29].

Microcerebrovascular endothelial cells express receptors that enable them to respond to systemic inflammation, such as the cytokines interleukin-1β (IL-1β), [30–32] interleukin-6 [33], tumor necrosis factor-α [34], and the pro-inflammatory molecule cyclophilin [35]. They also express Toll-like receptors (TLRs), e.g., TLR2, TLR3, TLR4, and TLR6, which are pattern recognition receptors for molecules derived from pathogens, namely, lipoteichoic acid from Gram-positive bacteria, double-stranded RNA from viruses, LPS from Gram-negative bacteria, and diacyl lipopeptides from Mycoplasma respectively [36]. TLR4 requires the coreceptor CD14, which is also expressed by cerebral endothelial cells [37].

### Basement membrane

The basement membrane at the BBB is an extracellular amorphous but highly organized matrix of four main types of structural proteins: collagen IV family proteins, nidogens, heparan sulfate proteoglycans such as perlecan, and laminins (Fig. 3). It is composed of two juxtaposed endothelium- and astrocyte (glial)-derived layers that differ in composition, especially with respect to laminin content, such that the endothelial layer contains laminin-411 and laminin-511, while the glial layer contains laminin-111 and laminin-211 (Fig. 3) [38]. A potential perivascular space is present between these two layers, where CD163-positive macrophages physiologically reside, and leukocytes sometimes congregate during inflammatory brain diseases. CD163-positive perivascular macrophages perform a scavenging function at the BBB [39] and express surface molecules that enable them to respond to systemic inflammation: scavenger receptor A types I and II [39, 40], mannose receptors [41], DC-SIGN [42], CD14 [43], CD18/CD11b/c [44], and IL-1β [45]. They also express antigen presentation molecules such as major histocompatibility complex type II, B7‐2, and CD40 [42]. While they play a negligible role in transducing an inflammatory message across the BBB compared to that of endothelial cells [46, 47], they perform an important role presenting antigen to lymphocytes crossing the BBB [48, 49].

The basement membrane plays an important role in determining BBB permeability to cells. Cellular migration occurs at capillaries and especially at postcapillary venules. The endothelial layer is characterized by areas of laminin 411 and 511 colocalization alternating with laminin 511-low areas, called low expression regions. These areas act as exit points for T-cell extravasation in postcapillary venules [50], since lymphocyte surface integrin α6β1- and αvβ1-mediated interaction with laminin 511 would otherwise hold cells in check [51]. After traversing the endothelial layer of the basement membrane, leukocytes tend to be held up in the perivascular space [52] and may survey the perivascular space at length [53]. They require an activation step in the perivascular space before they can proceed to cross the glial layer; [48] this passage is enabled by selective cleavage of dystroglycan, a transmembrane receptor that anchors astrocyte endfeet to the glial basement membrane by focal MMP-2 and MMP-9 action [54]. Hence, the glial-derived layer of the basement membrane presents a more formidable barrier to infiltrating leukocytes than its endothelial counterpart.

### Pericytes

Pericytes are neural crest-derived cells embedded within the endothelial basement membrane. Although pericyte coverage of the endothelium is ~40% [55], there is an intimate association between pericytes and endothelial cells [56] such that each pericyte forms thousands of cytoplasmic extensions into endothelial cell invaginations—the molecular components and functional implications of this anatomical arrangement are currently unknown. The pericyte-to-endothelial cell ratio in the brain, the value of which varies between 1:1 [57] and 1:3 [58], is the highest such ratio in the body.

Pericytes play two major roles at the BBB, which may affect the passage of solutes and cells into the brain. The first is the maintenance of BBB integrity by inhibition of transcytosis and by induction of endothelial cell expression of tight junction proteins [59, 60] and major facilitator domain-containing protein 2A (MFSD2a), a docosahexaenoic acid transporter [61]. The second is the regulation of capillary diameter and cerebral blood flow [62, 63]. The passage of substances into the brain parenchyma in terms of the amount of substance per unit gram of brain tissue depends on two main factors: the permeability characteristic of the BBB and the surface area of the BBB available for exchange, which together form the permeability surface product [9]. At low levels of permeability at the BBB, changes in cerebral blood flow have negligible impacts on the absolute amounts of circulating substances entering the brain, as predicted by the Renkin–Crone equation [64, 65], since permeability is the limiting factor [66]. However, if the substance is very BBB permeable by its nature or the BBB becomes highly permeable during pathology, cerebral blood flow starts to increasingly play a role. Hence, by regulating the BBB surface area and blood flow, pericytes control barrier function in a region-specific manner.

### Astrocytes

Astrocytic foot processes form the innermost layer of the BBB. They interdigitate and overlap, leaving no gaps between them so that the coverage is near total [55]. The clefts between apposed astrocyte endfeet are not sealed by tight junctions and therefore provide no absolute barrier to markers as large as horseradish peroxidase (44 kD) [67]. However, a closer study has revealed a heterogeneous barrier that impedes molecules as small as a 500 dalton fluorophore in some areas while allowing free passage in other areas [68], overall presenting measurable resistance with a diffusion coefficient roughly one order of magnitude lower than that in the brain parenchyma [15]. Moreover, astrocytes control BBB integrity remotely. For example, they produce angiotensinogen, which, after cleavage to angiotensin II, binds to type 1 angiotensin receptors on BBB endothelial cells; this triggers threonine phosphorylation and organized recruitment of occludin at tight junctions [69]. Astrocytes also produce angiopoietin-1, which acts at tie-2 receptors on endothelial cells [70] to promote tyrosine dephosphorylation of occludin, stabilizing tight junctions [71]. Another protein astrocytes produce is sonic hedgehog, which interacts with its receptor Patched-1 on endothelial cells to maintain a transcriptional program including all three molecular components of tight junctions [72]. The overall role of astrocytes at the BBB is controversial; for example, in one study, laser ablation of astrocytic endfeet in mice did not affect permeability to molecules in the range of 4–70 kD [73]; however, this study addressed a time window of only a few hours before neighboring astrocytes contributed new astrocytic endfeet.

Astrocytes at the BBB express aquaporin-4, a water channel that allows bidirectional water transport. Aquaporin-4 expression at the BBB changes during systemic inflammation [74] and in neuropathologies that are highly responsive to systemic inflammation, including conditions such as Alzheimer’s disease and multiple sclerosis [75]. The astrocytic pool of aquaporin-4 regulates water flux across the BBB, and the precise contribution depends on brain health status, mediating efflux of water in the healthy state and vasogenic edema and mediating influx of water in cytotoxic edema (see Box 1). During systemic inflammation, for instance, in rats challenged with intraperitoneal LPS, an increase in astrocytic aquaporin-4 expression and water permeability was observed in the absence of vasogenic edema (the latter being measured by brain water content) [74]. This is one illustration of the fact that an increase in BBB permeability does not necessarily equate to edema (e.g., if drainage pathways are unimpeded during vasogenic edema, or in the setting of cytotoxic edema), although vasogenic edema always implies a high BBB permeability. This explanation starts to provide some clarity to the controversy as to whether increased BBB water permeability and vasogenic edema are one and the same thing. An increased flux of water across the BBB during states of increased permeability is not accounted for by current kinetic tracer modeling of BBB permeability, which may lead to underestimation of clearance parameters such as Ktrans; this requires further study.

In summary, while the role of endothelial cells in BBB function has long been established, the most important recent studies have highlighted the roles of pericytes in BBB development, maintenance, and function [59–63]. The glycocalyx is the least-studied component of the BBB, and further work is required, particularly with regard to developing methods to measure its integrity in vivo, such as sidestream dark-field imaging [76]. There are some correlations between systemic glycocalyx thickness and the plasma concentrations of glycocalyx degradation markers [77]. Comparative studies of the brain and systemic glycocalyx are needed to identify specific molecular components of the BBB glycocalyx, the plasma concentrations of which may serve as markers of BBB integrity.

Box 1 Water permeability at the BBB: opposing roles of aquaporin-4 in different situations **Healthy state**

In the absence of neuropathology, aquaporin-4 appears to predominantly mediate the efflux of water. Mice with glial-conditional aquaporin-4 deletion had a higher brain water content than their wild-type littermates [168]; brain water content may be intracellular or extracellular. α-Syntrophin knockout mice whose aquaporin-4 cannot be anchored to the membrane displayed swollen astrocytic endfeet [169], and aquaporin-4-deficient mice had increased extracellular space [170]; hence, aquaporin-4 has a physiological role in the control of water homeostasis in both astrocytic intracellular and extracellular compartments.

**Pathological state: cytotoxic edema**

In pathological states accompanied by cytotoxic edema, water accumulates in the intracellular compartment, which is driven osmotically by reduced plasma osmolality in water intoxication and by increased intracellular osmolality due to Na^+^/K^+^ pump failure in ischemic stroke, hypoxia, and predominantly ischemic traumatic central nervous system injury. In these situations, aquaporin-4 has the opposite effect to the healthy state, mediating influx of water into astrocytes, since its knockout [171, 172] or pharmacological relocalization away from the cell membrane [173] improved or prevented edema. The mice with the glial-conditional aquaporin-4 deletion mentioned above were found to have less edema during water intoxication than their wild-type littermates [168], demonstrating how the direction of water flux changes between health and disease.

**Pathological state: vasogenic edema**

Vasogenic edema is accompanied by increased BBB permeability (although increased BBB permeability does not necessarily result in vasogenic edema—see the text). In sharp contrast to cytotoxic edema, aquaporin-4 has a protective role, mediating water efflux during conditions associated with vasogenic edema such as central nervous system injury or contusion [174], brain infection [175], and cancer [176].

## BBB responses to systemic inflammation

During systemic inflammation and/or infection, a plethora of circulating soluble inflammatory mediators may influence the BBB, as exemplified by the fact that serum from LPS‐treated mice, compared to serum from vehicle‐treated mice, compromised the integrity of an in vitro BBB model (as measured by the transendothelial electrical resistance of cultured primary mouse brain microvascular endothelial cells) [78]. However, other important effects occur within the circulation in response to systemic inflammation, including increases in the numbers of circulating leukocytes and their migratory potential, and circulatory physiological changes, as shown in Box 2.

**Fig. 4:**
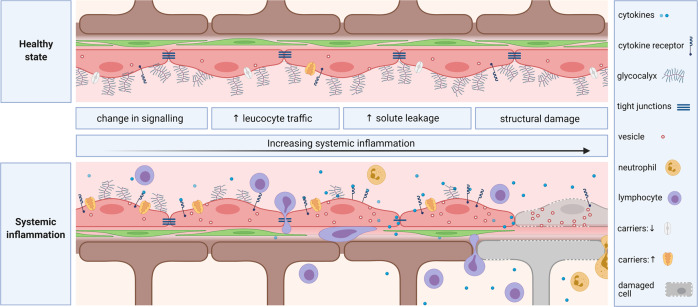
Vascular blood–brain barrier in health (upper panel) and during systemic inflammation (lower panel). The figure is divided into four vertical sections corresponding to the four types of BBB responses to increasing levels of systemic inflammation described in the text. In the first vertical section on the left, changes in signaling are exemplified by up- and downregulation of carriers and receptors. This is followed by increased cell and solute trafficking across the BBB, with enhanced transendothelial vesicular transport and tight junction breakdown, in the second and third vertical sections. A rolling lymphocyte has adhered to the endothelium, after which diapedesis into the potential perivascular space occurs, where the lymphocyte can crawl (step 1) or penetrate the glia limitans (step 2) to enter the brain parenchyma. The fourth vertical section illustrates structural damage to various components of the BBB, including the glycocalyx, basement membrane, endothelial cells, pericytes, and astrocytic endfeet

The BBB may react to systemic inflammation in several ways, although a limited number have been identified thus far. In order of degree of structural disruption to the BBB, these include changes in signaling, enhanced cellular traffic, an increase in solute permeability and direct damage (Fig. 4), which will be discussed in this section.

Box 2 Changes in systemic physiological parameters and circulating mediators during inflammation with the potential to affect blood–brain barrier function **Systemic physiological parameters**

- Hypotension with decreased cerebral blood flow
- Loss of cerebral autoregulation
- Hypoxia
**Immune cells**
- Natural killer cells
- Neutrophils
- Monocytes
- Lymphocytes
**Proinflammatory substances**
- Pathogen-associated molecular pattern molecules (e.g., LPS, viral nucleic acids)
- Cytokines (e.g., TNF-α, IL-1β, IL-6, IFN-γ)
- High-mobility group box‐1
- Cyclophilin A
- Sphingosine
- Complement components
- Kinins
- Prostaglandins
- Serotonin
- Histamine
- Arachidonic acid

### Changes in signaling

The normal physiological response of the cerebral endothelium to systemic inflammation is nondisruptive, achieving immune-to-brain signaling without invoking changes in permeability or cell trafficking across the BBB. It consists of a well-choreographed sequence of molecular events that transduce the systemic inflammatory message into changes in neuronal activity, leading to sickness behavior and protection of the host. Intravenous IL-1β challenge activated cerebral endothelial cells (as indicated by expression of the master transcription factor c-fos), before activation of the neighboring brain parenchyma [79], demonstrating that BBB activation is an intermediate step. Intraperitoneal injection of LPS resulted in upregulation of the mRNA expression of the cytokine-responsive nuclear transcription factor IκBα in the endothelium and activation of neurons, as indicated by c-fos mRNA expression; only the latter was inhibited by indomethacin, suggesting that the first prostaglandin-independent step is followed by a second prostaglandin-dependent step [80]. Further investigation has shown that endothelial-specific knockdown of IL-1R1 in mice blocked cyclooxygenase-2 expression in brain endothelial cells, neuronal activation as assessed by c-fos, fever, and locomotor hypoactivity, all of which are usually induced by circulating IL-1β [81]. Similar findings have been observed upon endothelial-specific knockout of key molecules in the signaling cascade: myeloid differentiation factor 88 (MyD88), an essential component of the intracellular IL-1R1 signal transduction cascade that leads to activation of a transcriptional program driven by the transcription factor NF-κB [47], and two prostaglandin E_2_ synthesizing-enzymes, cyclooxygenase-2 and microsomal prostaglandin E synthase 1 [82]. Based on these and other studies demonstrating this phenomenon, it is widely held that endothelial cells can signal an inflammatory message across the BBB in the absence of measurable changes in solute permeability by detecting IL-1β in the blood and synthesizing prostaglandin E_2,_ which then diffuses into the parenchyma to engage with cognate prostaglandin receptors on neurons and glia to induce sickness behavior.

Another example of immune-brain signaling across the BBB that is separate from BBB permeability relates to insulin. Systemic inflammation induced by intraperitoneal LPS increased the uptake of insulin into the brain independent of BBB disruption [83] through a mechanism involving posttranslational modulation of endothelial and inducible nitric oxide synthase enzyme activity levels [84]. High insulin levels resulted in decreased feeding, loss of weight, and changes in cognition [85], which are reminiscent of sickness behavior.

P-glycoprotein is a transporter at the BBB that has long been known to mediate brain-to-blood efflux of drugs and xenobiotics but was recently found to regulate endogenous steroid levels in the brain such that loss of P-glycoprotein results in increased brain aldosterone and anxiety-type behavior [86]. Since P-glycoprotein expression is reduced during systemic inflammation [87], this is another way by which changes in signaling at the BBB can mediate behavioral changes.

### Enhanced cellular traffic

Even in the absence of pathogen neuroinvasion or other neuropathology, systemic inflammation stimulates leukocyte passage into the brain. A low level of T cell immunosurveillance occurs in the healthy brain [2], which excludes B cells and innate immune cells [88]. However, systemic inflammation increases lymphocytic trafficking [89], and the influx of natural killer cells [90], neutrophils [91], and monocytes [92] begins to accompany elevated degrees of inflammation. Leukocyte entry into the brain is a highly coordinated process involving multiple sequential steps: rolling, firm adhesion, and diapedesis (Fig. 4). As discussed above, complete diapedesis is a two-step process involving a first step of transmigration across the endothelium and its basement membrane into the perivascular space (second interendothelial junction, lower panel, Fig. 3) followed by a second step during which the glial basement membrane is traversed (third interglial junction, lower panel, Fig. 3). Intravital microscopy studies in mice after cecal ligation and perforation to induce sepsis have captured increases in rolling and adhesion of leukocytes to the brain endothelium that are followed by infiltration [93]. Several changes at the BBB occur to enhance this process during systemic inflammation, including degradation of the glycocalyx [19, 21–23] and endothelial upregulation of E/P-selectins [94–96] (which mediate rolling), chemokines such as CCL2 [97] and integrin ligands such as ICAM-1 [91] (which mediate adhesion). Under inflammatory conditions, the cytokines tumor necrosis factor-α, IFN-γ, and interleukin-17 (IL-17) induce focal MMP-2 and MMP-9 activity at the BBB, which promotes chemokine-induced leukocyte migration through the basement membranes, especially the glial limitans [54, 98]. Endothelial sphingosine-1-phosphate receptor 2 also plays an important role, since experiments with knockout mice and pharmacological agonism have demonstrated that signaling at the receptor increased BBB solute permeability and promoted neutrophil infiltration via endothelial E-selectin expression [99].

### Solute permeability

Emerging evidence suggests that compensatory changes occur at the BBB during the initial stages of systemic inflammation that prevent BBB disruption, such as increased solute permeability. In one study, in mice treated for seven days with intraperitoneal LPS, microglial dynamics were visualized daily with in vivo two-photon imaging and immunoelectron microscopy. Microglia migrated toward blood vessels in response to the release of the chemokine CCL5 from endothelial cells. They started producing claudin-5 (a tight junction protein), infiltrated their processes through the basement membrane and made claudin-5-immunoreactive contacts with endothelial cells. Early obliteration of vessel-associated microglia through the well-timed inducible genetic expression of diphtheria toxin was associated with increased BBB permeability to 10-kD dextran. However, once inflammation was sustained, this initial protective response was followed by a transformation of the vessel-associated microglia to a CD68-positive phagocytic phenotype with loss of BBB integrity [100].

A number of studies have shown that solute permeability at the BBB is increased during systemic inflammation or infection. A systematic review concluded that this is not a universal finding, with 60% of studies able to demonstrate an effect [9]. This controversy may be rationalized by considering the molecular sizes of the markers used to measure BBB permeability, the severity of the inflammatory stimuli, and the presence of pre-existing brain pathologies, among a number of other factors. A commonly used surrogate marker of permeability is the ratio of albumin (67 kD) in cerebrospinal fluid (CSF) to serum, expressed as a quotient. The CSF/serum quotient of albumin was found to increase during systemic inflammation, but only in the presence of CSF abnormality; [101] it is possible that leakage was not seen in the absence of neurological disease because albumin is too large a molecule. Fibrinogen is an even larger molecule (340 kD), and while its concentration in postmortem human brain tissue homogenate was found to be increased during systemic inflammation, the latter was more severe, since the patients had died of sepsis [102]. Markers with smaller molecular weights should be more sensitive than larger molecules to changes in BBB permeability [103] and would better emulate the passage of cytokines (5–20 kD), yet studies with markers in this range of molecular size have yet to be performed in the context of systemic inflammation.

The BBB leakage that occurs during systemic infection may be exclusively mediated through systemic inflammatory effects without direct infection of the cerebral endothelium or brain parenchyma. For example, an increase in solute permeability is commonly observed in studies during which systemic inflammation is modeled by noninfective inflammatory challenges, such as administration of the bacterial cell wall component LPS [104], the double-stranded RNA molecule poly (I:C) (to simulate viruses) [105, 106], and the cytokine tumor necrosis factor-α [107]. In a series of patients dying from COVID-19, magnetic resonance microscopy and immunofluorescence showed areas of microvascular injury and fibrinogen leakage in the brain in association with endotheliitis [108]. In these series, none of the tissue samples had detectable SARS-CoV-2, demonstrating that if severe enough, systemic inflammation may cause damage to the BBB. Some systemic autoimmune inflammatory diseases have also been associated with increased solute permeability at the BBB. One example in which this has been shown in humans using dynamic contrast-enhanced magnetic resonance imaging is systemic lupus erythematosus [109]. However in these autoimmune diseases it is unclear whether systemic inflammation causes BBB leakage or whether a parallel factor such as autoimmunity against the cerebral endothelium or brain antigens is involved. In a mouse model of rheumatoid arthritis, using immunization with collagen II, a sustained increased BBB permeability to sodium fluorescein was seen throughout the time course studied up to 100 days after immunization, suggesting a link with inflammatory arthritis rather than the initial immunization [110].

A number of mechanisms link systemic inflammation with tight junction disruption to explain solute leakage across the BBB. An important pathway is the disruption of tight junctions via MMPs induced by inflammation. An immunohistochemical study of brain tissue from patients dying with sepsis showed decreased levels or absence of the tight junction molecules occludin, ZO-1, and claudin-5 [111]. Tight junction disruption in human cerebral microvascular endothelial cells can be mediated by MMPs after LPS exposure in vitro [112]. After cecal ligation and perforation in rats, which induces a model of sepsis, Evans blue leakage was MMP2 and MMP9 dependent such that inhibition of these two enzymes could reverse the clinical features of sepsis-associated encephalopathy [113]. Another important pathway is a decrease in endothelial sphingosine 1–phosphate receptor 1 signaling due to lower circulating levels of sphingosine 1–phosphate 1 during sepsis [114]. Sphingosine 1-phosphate receptor 1 maintains the BBB by regulating the proper localization of tight junction proteins [115]. Electron microscopy studies following the fate of circulating colloidal iron oxide after systemic LPS challenge [116] and horseradish peroxidase after cecal ligation and perforation [117] have found evidence of increased macropinocytosis.

Increased BBB solute permeability during sepsis has clinical repercussions. Sepsis-associated encephalopathy manifests clinically as delirium, and in a large study, elevated circulating markers of endothelial activation (plasminogen activator inhibitor-1 and E-selectin) and blood–brain barrier disruption/neuropathology (S100B) during critical illness were associated with delirium [118]. However, although sepsis-associated encephalopathy may be associated with solute leakage across the BBB, the latter is not essential. In one set of experiments in rats with sepsis after cecal ligation and perforation, behavioral changes in keeping with sepsis-associated encephalopathy were observed in association with cortical hypoperfusion and microglial/astrocytic activation—but this occurred in the absence of immunoglobulin G leakage [119]. This underlines the multifactorial nature of encephalopathy, which encompasses systemic physiological disturbances as well as circulating proinflammatory changes, and indicates how the contribution of these factors may be variable, even in animal studies.

Hypoxia is an important systemic factor present during sepsis, which may itself affect BBB function. An in vitro study using primary bovine cerebral microvascular endothelial cells showed that hypoxia (1% oxygen for 1 day) induced a 2.6-fold increase in sucrose permeability accompanied by alterations in occludin, ZO-1, and ZO-2 protein localization, suggesting a perturbation of tight junction complexes [120]. A similar phenomenon was observed in mice exposed to hypoxia (8% oxygen) for 4 days [121]. Exposure of primary human cerebral microvascular pericytes to hypoxic culture conditions (1% oxygen for 48 h) resulted in a 4.3-fold elevation of the shed soluble form of platelet-derived growth factor receptor β in the culture medium [122]. Interestingly, microglia protect against hypoxia-induced BBB disruption by aggregating around leaky vessels; their pharmacological depletion with the colony-stimulating factor-1 receptor inhibitor PLX5622 markedly increased hypoxia-induced BBB leakage [121].

### Direct damage

While changes in signaling, cellular trafficking, and solute permeability are commonly observed during systemic inflammation or infection, direct damage to the endothelium may occasionally occur. One example is COVID-19, caused by SARS-CoV-2. Electron microscopy of postmortem tissue from COVID-19 patients has demonstrated the presence of viral inclusion structures in systemic endothelial cells, and histological assessment has shown inflammatory cell infiltration close to the endothelium and endothelial apoptosis [123]. Another example is anti-CD19 chimeric antigen receptor T-cell immunotherapy for refractory B-cell malignancies. This treatment can cause serious neurotoxicity with a delayed onset of 5 days, known as immune effector cell-associated neurotoxicity syndrome, which is characterized by headache, delirium, aphasia, seizures, and other neurological deficits that progress to cerebral edema, coma and sometimes death [124]. There is evidence of BBB leakage to albumin and cells as well as endothelial cell destruction [125]. Anti-CD19 chimeric antigen receptor T cells recognize and destroy CD19-expressing pericytes, an unforeseen off-target effect, which leads to BBB leakage [126].

In summary, the most important advance in recent years has been the increasing recognition that systemic inflammation is associated with high BBB permeability, not only in animals but also in humans [101, 109]. Important next topics to address include (1) the regional susceptibility in terms of neuroanatomical areas and (2) whether this increased BBB permeability causes any transient or lasting clinically relevant pathology rather than being purely associative, which would make this pathway a therapeutic target.

## Moderators of the BBB response

**Fig. 5:**
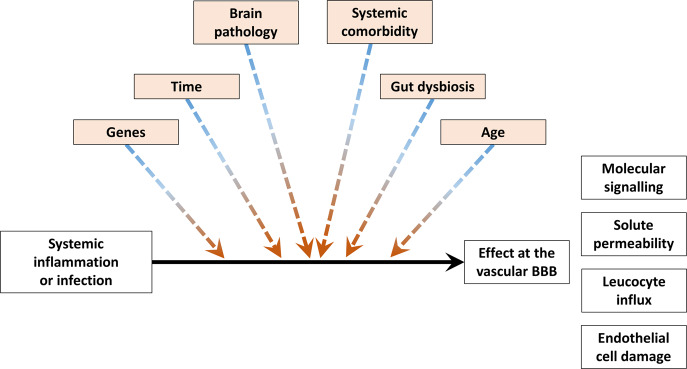
Moderators of the blood–brain barrier response to systemic inflammation and infection. Moderators are highlighted in light orange and have colored arrows. Stratifying on these moderators or accounting for them as covariates will become important in future experimental or observational studies of BBB permeability

The above section discussed in detail the cellular and molecular *mediators* of the BBB response to systemic inflammation. The causal relationship between the mediators and the BBB response may vary depending on the levels or magnitudes of *moderators*, such as genetics, age, pre-existing brain pathology, comorbidity, and time. This is important since moderators must be included in statistical analyses as additional covariates in regression analyses or factors in analyses of covariance with interaction effects (Fig. 5).

### Genetics

Genetics, including sex, play an important role in determining baseline BBB permeability; this is intuitive since variations in the genes coding for BBB proteins may result in changes in BBB structure and function. There are several rare genetic mutations causing neurological disease that, on further investigation, have been found to cause a constitutively leakier BBB. One example is the major facilitator superfamily domain-containing 2a (Mfsd2a), gene, mutations in which cause a lethal microcephaly syndrome [127]; this gene codes for a docosahexaenoic acid transporter that suppresses caveolae-mediated transcytosis in microcerebrovascular endothelial cells [61]. Another is the platelet-derived growth factor receptor β (PDGFR-β), mutations in which cause Fahr’s disease [128]; the normal protein is essential for pericyte recruitment and maintenance at the BBB [59]. More relevant to this review are common mutations that may explain individual variability in the BBB response to systemic inflammation. One example of a common protein regulating BBB permeability is apolipoprotein E (APOE), a cholesterol and lipid carrier. The *APOE* gene is polymorphic, with combinations of two single nucleotide polymorphisms resulting in three alleles (ε2, ε3, and ε4). In cognitively normal carriers of the *APOE* ε4 allele, BBB permeability as measured by the CSF/serum albumin quotient [129] and dynamic contrast-enhanced magnetic resonance imaging [130] is high compared to that in *APOE* ε2 or *APOE* ε3 carriers. One mechanism underlying this phenomenon is thought to be a loss of constitutive suppression of the cyclophilin A–nuclear factor-κB–matrix-metalloproteinase-9 pathway within pericytes that leads to MMP9-mediated degradation of capillary basement membrane and tight-junction proteins, and hence BBB leakage [131]. In keeping with this explanation, a high correlation between the CSF/serum albumin quotient, cyclophilin A and active MMP9 has been observed in the CSF of humans, with higher levels of all three in *APOE* ε4 carriers [129]. Other mechanisms may be involved. For example, APOE ε4 expression by pericytes was found to be associated with increased permeability and reduced levels of basement membrane collagen IV using an in vitro BBB primary mouse pericyte/endothelial cell coculture model [132].

There is evidence that genetics moderates the BBB response to systemic inflammation. For example, C57 versus CD1 and female versus male mice exhibited higher degrees of BBB disruption (as measured by brain uptake of a circulating radiotracer) after LPS challenge; some, but not all, of the gender effect may have been mediated by an increased cytokine response in females [133]. In another example, a sexually dimorphic and strain-specific role for sphingosine-1-phosphate receptor 2 was evident at the BBB, with higher expression in female SJL mice than in male SJL mice and C57BL/6 mice of both sexes [134]. Signaling at sphingosine-1-phosphate receptor 2 was associated with increased fluorescein permeability, luminal expression of the chemokine CXCL12, and diminished inflammatory cell infiltration [134]. Mice deficient in endothelial podocalyxin showed normal BBB permeability, but when stimulated with intraperitoneal LPS, there was a marked increase in 70-kD dextran leakage compared to the level in wild-type mice [135]. Of relevance to what we know about BBB genetics in humans, mice expressing human APOE ε4, compared to APOE ε3, exhibited greater BBB permeability to sodium fluorescein in the cortex after intraperitoneal challenge with LPS [136].

### Age

Numerous studies have confirmed that BBB permeability increases with age, using techniques such as CSF/serum albumin quotient measurement [137] and dynamic contrast-enhanced magnetic resonance imaging [138]. It is important to note that these techniques utilize tracers that do not require transporters, the expression of which decreases with age [24, 139]. A recent study in healthy male mice demonstrated a shift from receptor-mediated transport of plasma proteins (which decreases with age) to caveolar transcytosis (which increases with age) [140], thus confirming that the age-related increase in BBB permeability occurs for substances that use nonspecific pathways of transfer. Structural changes, such as those in tight junction expression, basement membrane thickness, or pericyte coverage, are not consistently seen to increase with age when considered singly [25]; however, their combination to various degrees is likely to be at least partially explanatory. Aging cells undergo senescence and acquire a senescence-associated secretory phenotype. This is linked to a transcriptional program geared toward the production of extracellular matrix proteases, cytokines, chemokines, and growth factors that stimulate leukocyte migration, activation, and infiltration [141]. Using single-cell RNA-seq, the unique transcriptional signature characteristic of cellular senescence was found in 10% of microvascular endothelial cells in the aged mouse brain at a biological age equivalent to 75 years in humans [142]. Cerebrovascular endothelial senescence has been found to be associated with BBB permeability in vitro using primary mouse brain endothelial cell and pericyte cocultures [143], and one mechanism is a decline in Sirtuin-1 expression, as shown in mice in vivo [144]. Experiments in mice have shown that aging is accompanied by an increase in expression of brain complement 3, which stimulates the receptor for the active signaling peptide of complement (C3aR) in the basolateral compartment of cerebral endothelial cells, resulting in the following effects on cellular and solute permeability: upregulation of the integrin ligand VCAM-1 enhancing lymphocyte entry and reduction of tight junctional protein expression with consequent increased solute permeability to a 65-85 kilodalton tracer [145].

There is evidence that age moderates the BBB response to systemic inflammation. Aged male Wistar rats rendered septic by the cecal ligation and perforation procedure demonstrated more profound BBB impairment than younger rats, and this affected both solute (Evans blue) and cellular (neutrophil) influx [146]. Higher-order interactions between moderators were evident between, for instance, sex and age in mice challenged with intraperitoneal LPS or vehicle; the increase in Evans blue BBB leakage with systemic inflammation was present in males only at a young age but present in both sexes at an older age [147]. This has been shown to be related to estrogen by combinations of ovariectomy and estradiol replacement [147].

### Brain pathology

Pre-existing neuropathology may render the BBB more sensitive to systemic inflammation. In mice expressing human-type amyloid-β precursor protein as a model of Alzheimer’s disease, BBB permeability to fluorescein isothiocyanate-labeled albumin after intraperitoneal LPS injection was higher than that in wild-type controls [148]. Similar observations have been made in animal models of ischemic stroke [149] and multiple sclerosis [150, 151], namely, that the effect of systemic inflammation on BBB permeability was more pronounced in the presence of central nervous system pathology. The combination of Alzheimer’s disease-like pathology (modeled by intracerebroventricular injection of oligomeric β-amyloid peptide) and cerebrovascular ischemia (modeled by striatal injection of endothelin-1) in rats caused greater vascular disruption than the same challenges individually [152].

Similar results are observed in humans. In a large study of 1273 human lumbar punctures in a general hospital setting, systemic inflammation as measured by C-reactive protein significantly predicted the CSF/serum albumin quotient in individuals with abnormal CSF findings but not in those without [101]. In a human postmortem study, fibrinogen measured by ELISA in brain tissue homogenate was used as a surrogate marker for BBB permeability; it was found to be higher in patients who were septic at the time of death than in those who were not septic, and this effect was more marked in patients with Alzheimer’s disease or vascular dementia compared to nondementia controls [102].

### Systemic comorbidity

A number of systemic comorbidities may affect BBB permeability, such as diet, alcohol consumption, and diabetes. A high-fat diet rendered the BBB more susceptible to the effect of age on immunoglobulin extravasation into the parenchyma, demonstrating an interaction between diet and age [153]. In primary human brain microvascular endothelial cell cultures, ethanol‐mediated oxidative stress led to decreased BBB integrity and facilitated monocyte transmigration [154]. In humans with type II diabetes, BBB permeability was increased as measured by signal intensity changes during contrast-enhanced magnetic resonance imaging; [155] evidence for causation has been established in rats with streptozotocin-induced diabetes [156, 157]. Whether alcohol intake or diabetes moderate the effect of systemic inflammation on the BBB remains to be shown. Multiple coexisting systemic inflammatory stimuli, such as coinfections, may interact with each other; for example, Evans blue leakage into the brain in mice inoculated intranasally with LPS and influenza A virus was much higher than the leakage seen with the single challenges [158].

### Gut microbiome

A firm link has been established between the gut microbiome and BBB integrity. Since it was first noticed that germ-free mice have a leakier BBB than mice with an intact microbiome, investigations have shown that short‐chain fatty acids such as butyrate induce tight junction formation at the BBB [159]. Butyrate and other short‐chain fatty acids, including propionate and acetate, are produced during the fermentation of complex plant-based polysaccharides by gut commensal bacteria. In rhesus monkeys, BBB permeability, as measured by the CSF/serum albumin quotient and dynamic contrast-enhanced magnetic resonance imaging, showed a clear increase after oral antibiotic treatment that was associated with a decrease in gut bacterial diversity and decreased levels of short‐chain fatty acids in fecal samples [160]. This pathway is being mapped out in humans. The human cerebral endothelium expresses free fatty acid receptor 3, which is the receptor for proprionate [161]. In a human microcerebrovascular endothelial cell line model of the BBB, propionate has been found to moderate the permeability-inducing effect of LPS [161].

### Time

The effect of systemic inflammation or infection on BBB integrity may vary as a function of time. Some of the BBB responses mentioned above may occur with a delay after the onset of the systemic challenge, especially if transcription and/or translation is required. The duration of systemic inflammation is important because sustained inflammation is more likely to overcome perivascular microglial protective capacity than transient inflammation, and loss of BBB integrity [100] and innate immune cell infiltration into the parenchyma may occur [92]. Changes to water permeability with cerebral edema may be complex depending on temporal sequencing of cytotoxic and vasogenic components; cytotoxic edema is not associated with increased BBB permeability, but if endothelial cells undergo swelling or there is bystander damage to the vascular wall, BBB integrity will be compromised, and vasogenic edema may develop [162].

In summary, important discoveries have been made in recent years relating to factors that moderate the BBB response to systemic inflammation. Perhaps the most important emerging factor is genetic background [129, 130], since this will allow stratification in clinical studies of BBB permeability. To date, studies have taken a candidate gene approach. However, unbiased studies are needed to determine the genetic determinants of BBB permeability since these determinants may modulate the BBB response to systemic inflammation. More studies are needed to find out whether, in progressive neurological diseases such as Alzheimer’s disease [148] and multiple sclerosis [150, 151], the increased susceptibility to BBB leakage during systemic inflammation contributes to neurodegeneration. If this is the case, the molecular pathway underlying BBB responsiveness to systemic inflammation can be therapeutically targeted to break the vicious cycle and slow progression.

## Conclusions

The BBB is a highly regulated interface between the blood and brain with a primary function to protect central neurons while signaling the presence of systemic inflammation and infection to the brain in order to enable a protective sickness behavioral response. Progress is being made in understanding the molecular and cellular determinants of the BBB in the healthy state and during the physiological response to systemic inflammation. With worsening degrees and durations of systemic inflammation, supraphysiological responses occur: the BBB undergoes an increase in solute permeability and lymphocyte trafficking, innate immune cell influx occurs, and endothelial cell loss may occasionally be seen. With these pathological changes at the BBB, encephalopathy manifests clinically. Several moderators may influence the direction and magnitude of the BBB response to systemic inflammation, including sex, genetic background, age, pre-existing brain pathology, systemic comorbidity, and gut dysbiosis. Further study is required to improve the tools that can be used to measure the BBB response to systemic inflammation and to advance our understanding of the genetic, system-level, cellular and molecular mediators and moderators of the BBB response. This will help explain the heterogeneity observed in animal and human studies.
